# Chemo-Sensory Markers for Red Wine Grades: A Correlation Study of Phenolic Profiles and Sensory Attributes

**DOI:** 10.3390/foods14173047

**Published:** 2025-08-29

**Authors:** Na Xu, Yun Wu

**Affiliations:** College of Food Science and Pharmacy, Xinjiang Agricultural University, Urumqi 830052, China; xuna9699@163.com

**Keywords:** wine, grade, phenolic substances, colour, taste

## Abstract

To reveal the characteristic physicochemical indicators of wines of different quality grades and explore their feasibility as auxiliary indicators for grading, 23 wines from the Manas subregion of Xinjiang were used as test materials. Sensory evaluation, colour difference analysis, and electronic tongue technology were employed, combined with nontargeted metabolomics and quantitative analysis, to analyze differences in phenolic compounds, colour parameters, and taste characteristics among wines of different grades. Finally, a quality evaluation model for Cabernet Sauvignon wine was constructed using partial least squares regression (PLSR). The results revealed significant differences in the L* values, a* values, and C*ab values among wines of different grades. Grade A wines presented lower L* values, higher a* values, and higher C*ab values, indicating lower brightness, deeper red tones, and higher saturation. Taste characteristic differences were primarily manifested in Grade A wines, which have higher acidity, astringency, bitterness, and richness but exhibit lower bitterness aftertaste and astringency aftertaste. The results of the quantitative analysis and correlation analysis indicate that the differences in sensory characteristics among different grades of wine stem from variations in their polyphenolic compound contents. The higher anthocyanin content in Grade A wine is associated with higher a* values; higher flavonoid content is closely related to higher astringency and bitterness values; and lower flavanol content is associated with lower bitterness aftertaste and astringency aftertaste values. The PLSR model results indicate that when sensory characteristic parameters and phenolic compound content are used as predictor variables (X) and grade is used as the response variable (Y), the PLSR model has a calibration set R^2^ = 0.97 and a validation set R^2^ = 0.92, the calibration set RMSE is 0.13, and the validation set RMSE is 0.25. The model demonstrates good fitting performance, establishing an objective method for evaluating wine quality that avoids evaluation errors caused by the subjective factors of winemakers and tasters. This study is the first to conduct a comprehensive evaluation of the sensory characteristic and chemical components of three grades of wine, providing data support and theoretical references for the improvement of wine quality evaluation systems.

## 1. Introduction

For a long time, the evaluation of wine quality has relied primarily on the sensory assessments of experienced professional wine tasters. Owing to individual differences among evaluators, including gender and experience, their evaluation results may be subject to subjective biases. Therefore, in recent years, the classification of wine quality on the basis of physicochemical indicators has become important for evaluating wine quality [1,2]. The comprehensive analysis and evaluation of red wine quality primarily consists of quality factors based on sensory attributes such as colour, aroma, and taste [3]. Among these factors, colour and taste represent two key factors in evaluating red wine quality. Colour influences the overall quality of wine; provides important information about wine type, age, and defects; and serves as a key reference indicator for consumers when purchasing wine [4,5]. Various chemical components, including flavour-producing and aroma-producing substances, interact to form the sensory characteristics of wine, eliciting human sensory perceptions of taste, such as sour, sweet, bitter, salty, and umami [5,6].

Polyphenolic compounds are the primary factors influencing the colour, flavour, and sensory characteristics of wine, contributing to the perception of wine quality [7,8,9]. Flavonoids are typically classified into nonflavonoid compounds and flavonoid compounds. Flavonoid compounds are the primary phenolic compounds in grapes and are categorized into flavanols, flavan-3-ols (catechins), and anthocyanins [10,11]. Anthocyanins and their derivatives are key components of red wine, playing a major role in colour and contributing significantly to specific sensory attributes. The composition and content of anthocyanins significantly influence the colour characteristics and stability of wine. For example, anthocyanins with higher degrees of acetylation or methylation exhibit stronger photothermal stability [8,12], potentially conferring more persistent colour performance to high-grade wines. In recent years, CIELab uniform colour space parameters have been widely applied in the objective evaluation of wine colour performance, and their correlation with sensory scores has been validated in multiple studies. Marcel Hensel et al. [13] compared the correlation among the Glories method, CIELab, and the human perception colour space in the light red, high-saturation colour region and reported that CIELab is closer to human perception and that the use of CIELab is superior to the Glories method. Fan et al. [14] constructed colour distribution maps and brightness distribution maps on the basis of the CIELab colour space to visually represent wine colour, successfully revealing the colour characteristics of 10 wines. However, the current method evaluates wine solely on the basis of CIELab parameters, without considering that wine colour-related parameters can also serve as important indicators for evaluating and distinguishing wines.

Taste perception is also an important indicator influencing the overall quality of wine, and flavonoid compounds are the primary factors affecting taste perception. Flavonols in flavonoids can undergo polymerization with small-molecule proteins, affecting the mouthfeel of wine [15,16]. Other studies have shown that flavanol groups, including monomeric flavan-3-ol and its polymers (also known as condensed tannins or proanthocyanidins), are associated with bitterness, body, and astringency [17,18,19]. An electronic tongue is an artificial intelligence system that simulates human taste perception, and its core technology is based on lipid bilayer membrane sensors [20,21]. When food flavour compounds bind specifically to the sensor’s biomimetic interface, cross-membrane potential drift can be detected to analyze basic taste parameters such as astringency, acidity, and bitterness precisely, resulting in a unique digital taste profile [22,23,24]. Rachel et al. [25] demonstrated that the electronic tongue, as a detection tool, is more sensitive than human panels and does not suffer from sensory fatigue, making it highly useful for winemakers seeking additional instrumental methods to detect wine defects. Cai et al. [26] demonstrated that the system overcomes the subjective limitations of traditional sensory evaluation, achieving an acidity detection accuracy of ±0.02 and expanding the quantitative detection range of umami compounds (monosodium glutamate) to 10–1000 ppm.

Currently, scholars both domestically and internationally have studied the relationships between wine colour and taste and phenolic compounds from various perspectives. However, there is a lack of in-depth exploration into the systematic associations among wine quality grades, colour, taste parameters, and phenolic compound composition. Therefore, in this study, the colour, taste parameters, and phenolic compound composition of wines of different quality grades in the Manas small wine region are measured. Various chemical analysis methods and instruments are used to analyze and determine the quality factors in wine, serving as a reference to reflect the sensory characteristics and quality levels of wine. The aim of this study is to identify indicative physicochemical indicators of the characteristics of wines of different quality grades in the Manas subregion and explore their feasibility as auxiliary grading indicators, thereby providing data support and theoretical references for the improvement of wine quality evaluation systems.

## 2. Materials and Methods

### 2.1. Materials and Reagents

This study included 23 samples of wine, all sourced from the Xinjiang Manas subregion by Xinjiang Niya Wine Co., Ltd. The vintages covered were 2019 (19), 2021 (21), 2023 (23), and 2024 (24); all wine samples were made from Cabernet Sauvignon. According to the scoring table of the Chinese Wine Evaluation System, the four vintage samples were scored separately, including 10 points for colour-related indicators, 30 points for aroma-related indicators, 50 points for taste-related indicators, and 10 points for overall evaluation, with a maximum score of 100 points. The tasting panel consisted of 12 experts certified as national wine tasters, and the evaluation was conducted in a standard sensory analysis laboratory. All samples were randomly coded by three individuals and presented in a completely random order to ensure that production information was completely concealed during the evaluation process. The final evaluation scores are expressed as the arithmetic mean (mean ± SD) of the 12 experts’ scores. A total score of ≥90 points was classified as Grade A wine, a total score of 80–89 points was classified as Grade B wine, and a total score of <80 points was classified as Grade C wine. The final wine samples were numbered according to the sequence of vintage (19, 21, 23, 24), grade (A, B, C), and serial number (1, 2, etc.) (e.g., 19-A-1 refers to the first Grade A wine sample from 2019).

Ethanol, phenolphthalein, citric acid, sodium citrate, sodium hydroxide, hydrochloric acid, sulphurous acid, anhydrous copper sulphate, resorcinol, methanol, ascorbic acid, acetone, sodium acetate, phosphoric acid, hypomethyl blue, and acetic acid (all analytically pure) were purchased from Tianjin Chemical Factory. The floridoid substances used for the quantification of floridoid substances, dimethyl floridoidin, formic acid, methanol, and acetonitrile were purchased from Sigma-Aldrich. The standards for the quantification of anthocyanin, dimethyl anthocyanin, formic acid, methanol and acetonitrile were purchased from Sigma-Aldrich.

### 2.2. Instruments and Equipment

T6 Ultraviolet spectrophotometer: Shanghai General Instrument Company (Shanghai, China); electronic tongue: INSENT Company, Kanagawa, Japan; FA2004 electronic analytical balance: Shanghai Shunyu Hengping Scientific Instrument Co. (Shanghai, China). EC-C18 column (150 × 3.0 mm, 2.7 μm), Agilent, Santa Clara, CA, USA; SG3200HBT ultrasonic cleaner: Shanghai Guantech Ultrasonic Instrument Co. (Shanghai, China).

### 2.3. Test Methods

#### 2.3.1. Determination of Basic Physicochemical Indices

The residual sugar, volatile acid, total acid, and alcohol contents and pH of the wine samples were determined with reference to GB/T 15038-2006 (General Methods of Analysis for Wine and Fruit Wine) [27].

#### 2.3.2. Determination of CIELab Parameters

The method described by LI et al. was referenced for this experiment [6]. After the wine samples were filtered through a 0.22 μm water-based filter membrane, a 2 mm diameter quartz cuvette with distilled water was used as the control. The absorbance was measured at wavelengths of 450 nm, 520 nm, 570 nm, and 630 nm using a UV-visible spectrophotometer, and the CIELab colour parameters of the test wine samples were calculated.

#### 2.3.3. Determination of Phenolic Content

The method described by Yang et al. [28], who employed high-performance liquid chromatography (HPLC) coupled with triple quadrupole mass spectrometry (QqQ) equipped with a Poroshell 120 EC-C18 chromatographic column, was referenced for this experiment. The samples were filtered through a 0.22 μm aqueous membrane filter, with an injection volume of 5 μL. Mobile phase A was a 0.1% formic acid aqueous solution, and mobile phase B was a 50/50 (*v*/*v*) acetonitrile–methanol solution containing 0.1% formic acid. The detector was set to multiple reaction monitoring (MRM) mode. The elution programmes for anthocyanins and nonanthocyanin phenols were set according to the literature [29].

Mass spectrometry was performed in positive ion mode with a spray voltage of 6 kV, an ion source temperature of 170 °C, a drying gas temperature of 330 °C, a flow rate of 14 L/h, a nebulizer pressure of 38 psi, and a detector in multiple reaction monitoring mode. Quantification was performed using the external standard method for relative quantification. Peak area integration with qualitative analysis using Masshunter software (version 10.0) as the basis for quantification, with linear fitting curves of the peak area and concentration from different gradient standards used for quantification. For anthocyanins, dimethylflavone-3-O-glucoside was used as the external standard. For flavanols, myricetin-3-O-glucoside was used as the external standard. For flavanones, catechin, epicatechin, and epigallocatechin were used as external standards. The concentrations of various substances in wine are expressed in mg/L.

#### 2.3.4. Determination of Taste Indices

The taste analysis instrument uses a Japanese custom electronic tongue equipped with five taste sensors, namely, AAE, CAO, CTO, COO, AE1, which are sensitive to umami, sourness, saltiness, bitterness, and astringency, respectively. Before wine samples are tested, the reference electrode and sensors must be activated for 24 h. A total of 40 mL of each wine sample is accurately transferred into the corresponding small beaker. First, the sensors in the prepared positive and negative cleaning solutions are cleaned for 90 s and then rinsed in the reference solution for 120 s until equilibrium is reached to obtain the reference solution potential. Finally, the sensors are immersed in each wine sample for 30 s to obtain the sample potential. Each sample is tested in quadruplicate, with the last three sets of data selected for analysis. Prior to formal data collection, the electronic tongue undergoes self-testing to ensure the reliability and stability of its output results, with the testing temperature maintained at approximately 20 °C. The taste perception output value is calculated using the potential difference. The electronic tongue’s electrical signal taste perception score is converted and output using the system’s built-in calculation method.

### 2.4. Statistical Analysis

Samples were assayed in three parallels and kept for ANOVA and Duncan’s test (*p* < 0.05). Data were processed using IBM SPSS Statistics software (version 27.0) and plotted using Graphpad Prism software (version 10.1.2) and Origin2024 software (version 2024). Quality prediction models were analyzed using The Unscrambler X (version 10.4).

## 3. Results and Discussion

### 3.1. Basic Physical and Chemical Indicator Analyses

As shown in Appendix A the alcohol content of the 23 wine samples tested ranged from 11.49 to 15.21 %vol. The reducing sugar content ranged from 3.58 to 5.90 g/L. The volatile acid content ranged from 0.43 to 0.77 g/L. The total acid content ranged from 5.18 to 6.34 g/L. pH values ranged from 3.59 to 3.96. There were no significant differences between the samples of different grades, and all met the requirements of GB/T 15037—2006 “Wine” [30].

### 3.2. Colour Parameter Analysis and Visualization Representation

The CIELab parameters quantify lightness L*, red/green components a* (a* > 0 indicates a red hue; a* < 0 indicates a green hue), yellow/blue components b* (b* > 0 indicates a yellow hue; b* < 0, blue hue), chroma C*ab, and hue h*ab and can objectively describe the depth, hue, and saturation of wine colour [31]. To eliminate the influence of ageing duration on wine colour changes between grades, wine samples from the same vintage were grouped separately for analysis.

**Figure 1:**
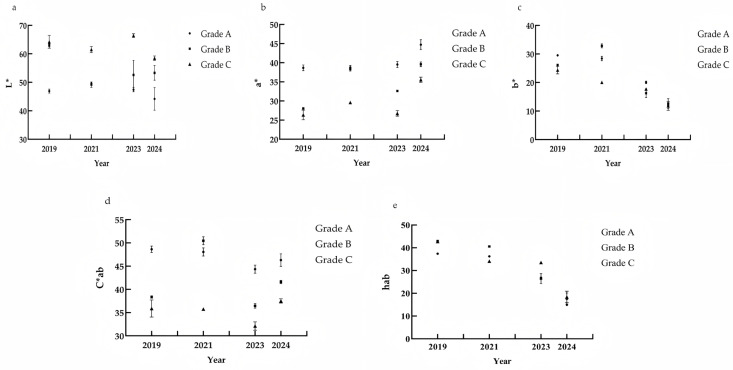
CIELab Colour parameters for wines lightness L* (**a**); red/green components a* (**b**); yellow/blue components b* (**c**); chroma C*ab (**d**); hue h*ab (**e**).

The lightness L* values of the wines are shown in Figure 1a. Among the four vintages, the L* values of Grade A wines were lower than those of Grade B and Grade C wines, indicating that Grade A wines have lower brightness and deeper, more intense colour. These findings also suggest that Grade A wines maintain better colour stability and can undergo slow browning after ageing. Figure 1b shows the changes in the a* values of wines of different grades across different vintages. The a* values of Grade A wine samples were greater than those of Grade B and Grade C wine samples of the same vintage across all four vintages, indicating that Grade A wines have stronger purple and red tones. The a* value is an important indicator of wine quality. This finding is consistent with the results of Parpinello et al. [32]. When the differences between the same grades across different durations are compared, the red hue a* value shows an overall decreasing trend. Compared with those of new wines, the samples aged for one year significantly decreased, and the values stabilized after the third year of ageing. Compared with Grade B and Grade C wines, Grade A wines presented a smaller decrease in a* values as the ageing time increased, demonstrating better retention of red hue and superior stability. The b* values of all the tested wine samples ranged from 10.24 to 32.82. There was minimal variation in b* values among different grades within the same vintage. However, as shown in Figure 1c, the b* values of the wine samples increased significantly with increasing ageing time. The b* values of the 2024 wine samples ranged from 10.24 to 14.43, whereas the b* values of the 2019 wine samples ranged from 23.43 to 29.53. This finding indicates that as the age of the wine increases, the yellow hue in the wine becomes more pronounced, and the degree of yellowing becomes more severe, which is consistent with the findings of Xie et al. [33]. Overall, the primary factor influencing the b* value of wine is the ageing time. The C*ab colour intensity parameter characterizes the degree of colour concentration, with higher values indicating more concentrated colour and higher colour saturation [31]. As shown in Figure 1d, the C*ab colour intensity values of the wine samples ranged from 32.11 to 50.47, indicating that the tested wine samples had high colour saturation, vibrant colours, and minimal variation. Among wines of the same vintage but different grades, the colour saturation of Grade A wine samples is generally slightly greater than that of Grade B and Grade C wine samples. However, when the wine is 3 years of age, Grade B wine has the highest colour saturation, but its yellow hue is prominent, which may have affected the overall sensory evaluation score. In young wines, the colour values of different grades show minimal difference, with a uniform and consistent distribution. As wine age increases, the C*ab values of Grade A samples are significantly greater than those of lower grades, suggesting that C*ab values may serve as an indirect indicator for assessing the quality of aged wines. One of the primary and most important characteristics of colour is hue (hab), which represents the overall tendency of colour and corresponds to the peak position of the absorption spectrum. The values 0° (or 360°), 90°, 180°, and 270° correspond to red, yellow, green, and blue hues, respectively [31], as shown in Figure 1e. The hue value is smallest in young wine, with the wine body exhibiting purple-red or ruby-red hues, which characterize the colour features of young red wine. As the wine ages, the value increases, and the wine body tends towards brick-red or terracotta-red hues, which are characteristic of aged red wine. Minimal differences in hue values are noted among different grades of the same vintage. In this study, it was observed that using hue values to distinguish different ageing times for Cabernet Sauvignon wines was more effective. This result is similar to those of previous studies that used hue angles to determine wine age [34].

Overall, Grade A wine samples presented relatively high a* and C*ab values across all four vintages, with purple and red tones contributing significantly to the colour of the wine. In addition, relatively low b* values indicate relatively few yellow tones; and relatively low L* values result in relatively low brightness, indicating a subtle lustre with high tonal depth.

**Figure 2:**
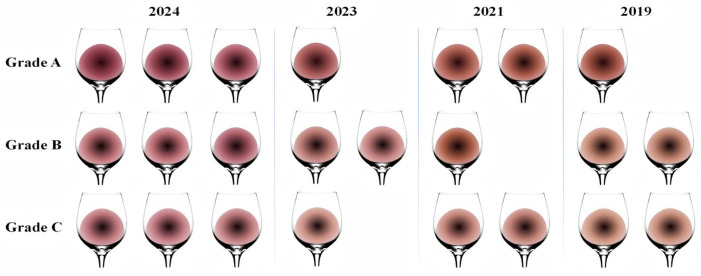
Wines feature colour.

Using ColorTell (https://www.colortell.com/colortool, accessed on 30 July 2025) professional colour analysis software, 23 wine samples were systematically positioned to obtain the L*, a*, and b* three-dimensional colour coordinates of each wine sample precisely under natural white light and a 10° observation angle. By establishing a wine colour evaluation system based on human visual perception attributes [7,35], this study simulated the visual colour profiles of wines of different grades over four years, as shown in Figure 2. The visualization analysis results indicate that, in terms of colour space distribution, Grade A wines from the four vintages present significant colour clustering characteristics, generally with lower brightness, deeper red tones, and a significant positive shift in a* values, indicating an overall rich, saturated deep red colour profile, whereas the colour evolution trajectories of wine samples from different vintages are clearly distinguishable. Young wine samples present a distinct purple-red hue. As the ageing time increased, the wine underwent slow oxidative reactions, with yellow tones gradually dominating, whereas the brightness increased, resulting in a brick-red colour with moderate saturation and a slight amber lustre. This aligns with CIELab system results. This gradual colour transition provides a visual and quantitative basis for assessing wine quality and determining ageing duration.

### 3.3. Analysis of Taste Characteristic Results

**Figure 3:**
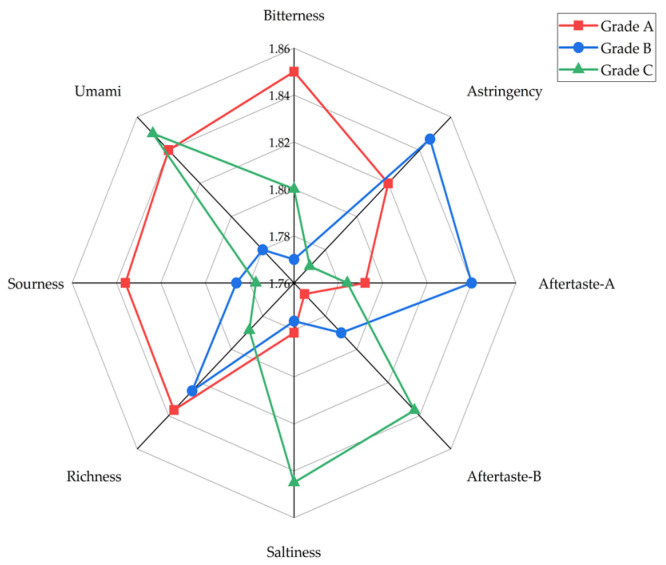
Electronic Tongue Radar Chart.

**Figure 4:**
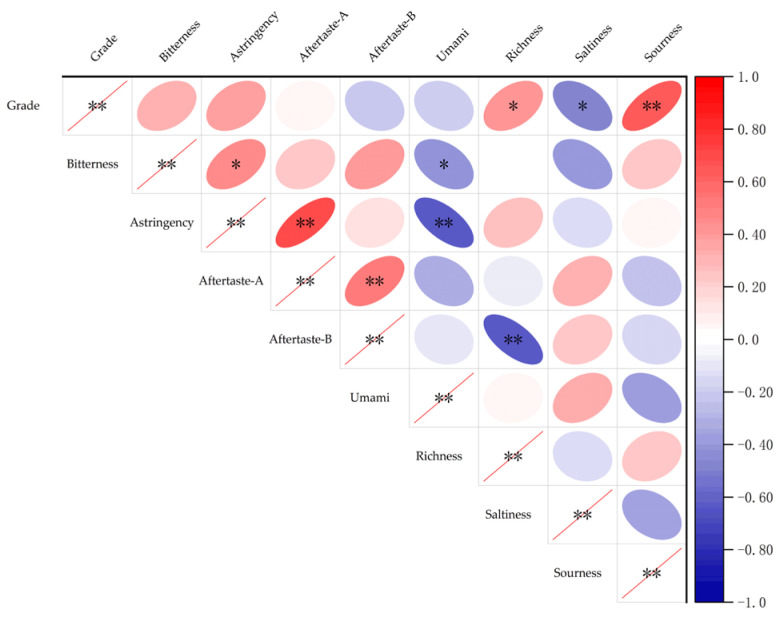
Correlation analysis of taste indicators. Note: ** means that the correlation between the two substances is highly significant, *p* < 0.01; and * means that the correlation is significant, *p* < 0.05.

The radar chart in Figure 3 shows the typical taste characteristics of different grades of wine samples as analyzed by the electronic tongue. Grade A wine samples exhibit balanced taste characteristics, with a distinct blend of astringency, bitterness, umami, acidity, and richness. Grade B wine samples have significantly greater astringency and astringency aftertaste response values compared with the other taste characteristics, resulting in a harsh, dry astringency that disrupts the wine’s overall harmony. Grade C wine samples, on the other hand, exhibit prominent saltiness, distinct bitterness aftertaste, and umami, with low astringency and acidity response values, resulting in a relatively bland overall taste lacking structural depth. Correlation analysis in Figure 4 reveals that the grade of wine was highly significantly positively correlated with acidity (*p* < 0.01), significantly positively correlated with richness (*p* < 0.05), and moderately positively correlated with astringency and bitterness. The correlations among taste indicators are as follows: bitterness and astringency are significantly positively correlated (*p* < 0.05) and significantly negatively correlated, respectively, with umami (*p* < 0.05); astringency is extremely significantly positively correlated with astringent aftertaste (*p* < 0.01) and extremely significantly negatively correlated with umami (*p* < 0.01); and bitter aftertaste is extremely significantly positively correlated with astringent aftertaste (*p* < 0.01) and extremely significantly negatively correlated with richness (*p* < 0.01). Pascual [36] and Gombau et al. [37] both studied the relationship between grape seeds and stems and the bitterness and astringency of wine and reported that grape seeds and stems are among the primary determinants of bitterness and astringency in wine. Therefore, on the basis of the results of this study, it is hypothesized that owing to the similarity in the sources of bitterness and astringency in wine and the chemical structures of their flavour compounds, bitterness and astringency coexist, and their intensities are positively correlated, exhibiting a synergistic expression mechanism. The negative correlation between bitterness, astringency, and umami may be due to the strong astringency of bitterness and astringency, which overshadows the smoothness of umami and inhibits its expression. Astringency is a common sensory quality in Cabernet Sauvignon wine, but appropriate bitterness can enhance the body and fullness of the wine [17,38]. The analysis results indicate that Grade A wine samples achieve a rich, multidimensional taste experience through the appropriate balance of astringency, bitterness, umami, and acidity. Research indicates that the final taste quality and style of wine are attributed to the interactions between phenolic compounds, making wine a unique and identifiable product [39]. Therefore, further research on phenolic compounds in wine and their correlation with sensory characteristics is necessary.

### 3.4. Analysis of Phenolic Compounds in Red Wine

Polyphenolic compounds in wine are the core substances responsible for the colour, antioxidant activity, and sensory characteristics of wine. By quantitatively analyzing anthocyanin and nonanthocyanin polyphenolic compounds in test wines, we can reveal the differences in flavonoid metabolism in wines and the mechanisms by which these differences affect wine quality.

#### 3.4.1. Analysis of Anthocyanin Content

Anthocyanins are the primary pigments responsible for the colour of red wine and play a significant role in the sensory quality of wine. Currently, hundreds of anthocyanins and their derivative pigments, which exhibit diverse structures and properties, have been identified and detected in various wines or model wines [33,40]. In this study, 23 wine samples were analysed, and all the samples were found to contain 15 anthocyanins. These 15 anthocyanins were classified into acetylated anthocyanins, acetylated anthocyanins, coumaroyl-acylated anthocyanins, anthocyanidins, anthocyanins, methyl anthocyanidins, methyl anthocyanins, and dimethyl anthocyanidins, as shown in Table 1. Among all the tested wine samples, dimethyl anthocyanins had the highest content, accounting for more than 80% of the total anthocyanin content, followed by Petunidin anthocyanins and Peonidin anthocyanins. The dimethylflavone B ring with the highest proportion contains three methoxy groups, which shift its maximum absorption wavelength towards purple-red [41]. Therefore, wines with high anthocyanin contents typically exhibit deep purple-red or ruby-red hues. An analysis of the anthocyanin content across wine samples revealed that the total anthocyanin content of all the tested wine samples ranged from 65.1 to 851.0 mg/L. The anthocyanin content varied significantly between vintages. Bimpilas et al. [42] reported that in red wine, the total anthocyanin content decreased nearly 10-fold over a one-year storage period, which was attributed to polymerization reactions and copigmentation, which is consistent with the results of this study. The total anthocyanin content of Grade A wines ranged from 90.8 to 851.0 mg/L, that of Grade B wines ranged from 86.3 to 716.2 mg/L, and that of Grade C wines ranged from 65.1 to 646.9 mg/L. Overall, the ranges for Grade B and Grade C are relatively close, whereas Grade A has a relatively higher total anthocyanin content. These findings indicate that high-quality wine has a relatively high anthocyanin content and that the anthocyanin content can shift the colour of wine towards red. Additionally, acylation enhances the redshift effect, increasing the intensity of the colour. This finding is consistent with the higher a* value observed in Grade A wines in the CIELab system.

#### 3.4.2. Analysis of Nonanthocyanin Phenolic Compounds

The concentrations of nonflavonoid polyphenolic compounds in the test wines are shown in Table 2. A total of five classes of flavanol compounds and six classes of flavanol compounds were detected. Among the flavanol compounds, the highest proportion was found in myricetin, followed by isorhamnetin, while kaempferol had the lowest proportion. Kaempferol compounds were detected in only the 2024 wine samples and one 2023 wine sample, with a concentration of only 0.1 mg/L in the 2023 wine sample. As the ageing time increased, the content of flavonoid compounds decreased, but the rate of decrease also gradually slowed. During the first year of ageing, the content of flavonoid compounds decreases significantly. However, in wines aged one year or more, the changes in flavonoid compound content stabilize. Among the four vintages, the total flavonoid concentration in Grade A wines was significantly greater than that in Grade C wines, with the most significant difference observed in the 2024 wine sample. Sensory parameter analysis revealed that Grade A wines presented relatively high astringency and bitterness values. The flavonoid compound content may influence the astringency and bitterness of wine, thereby affecting its overall quality.

Four monomeric flavanols (catechin, epicatechin, gallocatechin, and epigallocatechin) and two proanthocyanidins (B1 and B2) were detected in the wine. Among the flavanol compounds, catechins had the highest proportion, followed by epicatechins and proanthocyanidin B1, with epigallocatechin having the lowest proportion. As the ageing time increased, the total flavanol concentration gradually decreased, with the rate of decrease slowing over time, which was consistent with the trend observed for flavonoids. No significant differences in total flavanol concentrations were noted among different grade wine samples in 2024. However, in the other three years, Grade A wines exhibited significantly lower levels than Grade C and Grade B wines. Combined with sensory parameter analysis, Grade A wines presented lower bitterness and astringency aftertaste values, suggesting a correlation between flavanol compounds and bitterness and astringency aftertaste. Overall, nonanthocyanin phenolic compounds in wine are significantly influenced by grade and ageing time.

### 3.5. Correlation Analysis Between Sensory Indicators and Phenolic Substances

**Figure 5:**
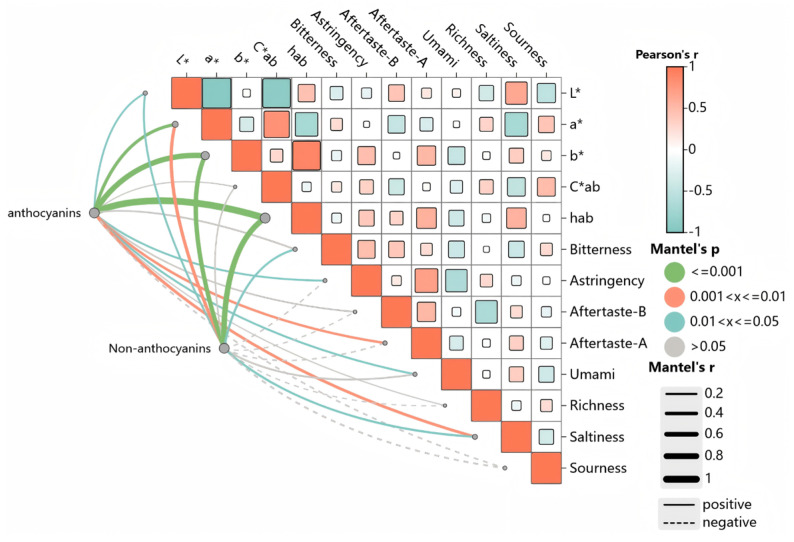
Interactive Mantel test correlation heatmap.

A Montel test correlation heatmap analysis was conducted on the colour parameters, taste characteristics, and contents of anthocyanins and nonanthocyanin compounds among the sensory attributes, as shown in Figure 5. The sizes of the squares in the figure represent the correlation coefficient values, with red squares indicating positive correlations and blue squares indicating negative correlations. The lines connecting the matrix show the associations between the sensory characteristics and phenolic profiles, with thicker lines indicating stronger correlations. Within the matrix, L* values showed significant positive correlations with hab, bitter aftertaste, and salty taste and significant negative correlations with a* values, Ca*b values, and sour taste. In addition, a* values showed significant positive correlations with Ca*b values and sour taste and significant negative correlations with hab values, bitter aftertaste, and salty taste, which is the opposite of L* values. Moreover, b* values were significantly negatively correlated with hab values, astringency, astringent aftertaste, and umami. C*ab was significantly positively correlated with acidity and significantly negatively correlated with saltiness, and hab was significantly positively correlated with astringent aftertaste and saltiness. These findings indicate that the stronger the red hue and the higher the saturation of the wine sample are, the stronger the acidity and bitterness of the wine body. It is hypothesized that a sensory interaction phenomenon may exist between the colour characteristics and taste characteristics of wine. Xia et al. [38] studied the effect of adding white grape seeds on the quality of red wine and reported that adding grape seeds could simultaneously improve the colour stability and astringency of red wine, which also illustrates the interaction phenomenon between colour and taste. In addition to the matrix, anthocyanin compounds presented extremely significant positive correlations with a* values, b* values, and hab values (*p* < 0.001); extremely significant positive correlations with astringent aftertaste and saltiness (*p* < 0.01); and significant positive correlations with L* values, astringency, and umami (*p* < 0.05). This finding indicates that sensory characteristic factors such as a* values, b* values, hab values, astringent aftertaste, saltiness, L* values, astringency, and umami undergo significant changes with variations in anthocyanin compounds. Anthocyanin compounds exhibit a weak negative correlation with acidity, indicating that changes in anthocyanin compounds have minimal impact on acidity. Nonanthocyanins exhibited a highly significant positive correlation with b* values and hab values (*p* < 0.001), and a significant positive correlation with L* values, bitterness, and saltiness (*p* < 0.05). These results indicate that nonanthocyanins significantly influence these sensory factors. Pauline et al. [43] reported that bitterness and astringency are positively correlated with the content of nonanthocyanin phenolic compounds, which is similar to the results of this study. On the basis of these findings, anthocyanin compounds primarily influence colour parameters and have relatively little effect on taste characteristics. Taste characteristics are associated with nonanthocyanin phenolic compounds, which also exhibit a secondary colour effect, influencing wine colour. This explains why the correlation between colour characteristics and taste characteristics may be attributed to the contribution of phenolic compounds.

### 3.6. Building a Quality Prediction Model for Wines of Different Quality Levels

**Figure 6:**
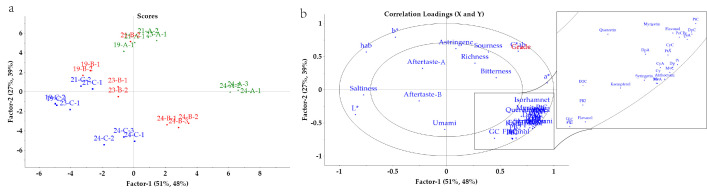
Partial Two-dimensional score plot (**a**) of PLSR and Two-dimensional load plot (**b**) of PLSR..

To demonstrate that both chemical and physical indicators in wine collectively influence its quality, a partial least squares regression (PLSR) analysis was conducted on chemical and physical indicators such as anthocyanins, polyphenolic compounds, colour, and taste parameters. Fifteen anthocyanins, five types of flavanol compounds, six types of flavanol compounds, five colour parameters, and eight taste characteristics were used as independent variables (X), whereas wine grade was used as the dependent variable (Y) to establish the relevant model. All the data were standardized prior to model construction. Figure 6a shows a score plot without outliers, where Factor-1 (51%, 48%) indicates that the first latent factor explains 51% of the variance in the predictor variables and 48% of the variance in the response variable. By moving from right to left along the plot, a trend is observed where wines of the same vintage transition from Grade A to Grade C, indicating that Grade A wines significantly differ from Grade C wines in terms of phenolic compound content, CIELab parameters, and taste characteristics. The changes in wine grade are closely related to phenolic compound content, CIELab parameters, and taste characteristics, with more pronounced differences observed within wines of the same vintage.

**Figure 7:**
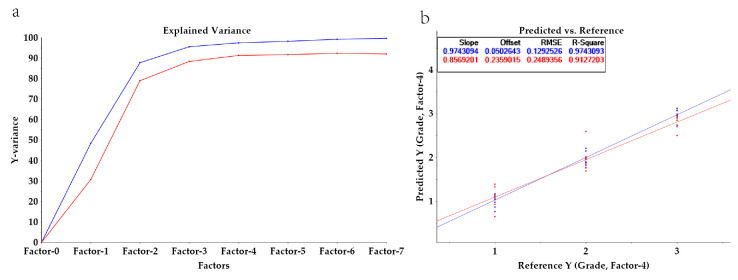
Explained Variance of PLSR model (**a**); PLSR predictive model (**b**).

Figure 6b presents a correlation loading plot, which shows the correlations between the predictor variables (X) and response variables (Y) and the latent factors Factor-1 and Factor-2 in the PLSR model. In the figure, both the predictor variables and response variables fall within two ellipses, indicating that the predictor variables and response variables are statistically significant. The latent factors Factor-1 and Factor-2 explain 78% of the predictor variables and 87% of the response variables, indicating that the model effectively explains these variables. The L* values, b* values, and hab values of the wine samples are all in the negative region of the *X*-axis. In contrast, the detected wine anthocyanin compounds, flavanols, and flavanones are all in the positive region of the *X*-axis. This indicates a negative correlation between phenolic compounds and the L* values, b* values, and hab values, whereas a strong positive correlation is noted with the a* and C*ab values along the positive half-axis. Additionally, bitterness, richness, acidity, and astringency are also in the positive half of the *X*-axis, showing a certain correlation with phenolic compounds, which is consistent with the results of the Montel test correlation heatmap. As shown in Figure 7a,b, the model achieves over 80% accuracy after the second principal component. After cross-validation testing, the PLSR model for wine grade has a calibration set R^2^ = 0.97, a validation set R^2^ = 0.92, a calibration set RMSE of 0.13, and a validation set RMSE of 0.25, demonstrating good model fit.

## 4. Conclusions

This study systematically analyzed the differences in colour parameters, taste characteristics, and phenolic compounds among Cabernet Sauvignon wines of different grades produced in the Manas region of Xinjiang. Through systematic analysis, we not only identified the distinguishing indicators of wines of different grades in this region but also explored the correlation between sensory characteristics and phenolic compound content.

The results revealed a relationship between phenolic compound content and sensory characteristics, indicating that the quality of wine is influenced by its phenolic compound content. Grade A wines presented lower brightness, a deeper red hue, and higher colour saturation, which was associated with higher anthocyanin content and the auxiliary colour effects of flavanols and flavanones. In terms of taste, differences in acidity, astringency, bitterness, astringent aftertaste, and bitter aftertaste were noted. Grade A wine presented more pronounced acidity, bitterness, and astringency, with lower astringency aftertaste and bitter aftertaste. This is related to the content of flavanol and flavanol compounds. Additionally, this study used different grades of Cabernet Sauvignon wine as samples and constructed a quality evaluation model for Cabernet Sauvignon wine based on sensory indicators and phenolic compounds, enabling a more comprehensive and integrated assessment of wine quality and, to some extent, avoiding evaluation errors caused by the subjective factors of wine tasters and winemakers.

In the future, we will systematically analyze volatile aromatic compounds in wines of different grades, elucidate the mechanisms underlying the correlation between aroma fingerprint profiles and sensory quality, and establish a multidimensional evaluation system for phenolic compounds, aroma, and taste.

## Figures and Tables

**Table 1:** The content of monomeric anthocyanins in wines.

Sample	Acylated Anthocyanins	Non-Acylated Anthocyanins	Coumarylated Anthocyanins	Delphinidin Anthocyanins	Cyanidin Anthocyanins	Petunidin Anthocyanins	Peonidin Anthocyanins	Malvidin Anthocyanins	Anthocyanins
2024									
24-A-1	308.0 ± 1.6 a	420.7 ± 0.3 a	122.3 ± 3.5 a	24.0 ± 0.3 a	3.4 ± 0.3 a	55.4 ± 2.2 a	47.3 ± 1.8 a	720.9 ± 5.1 a	851.0 ± 4.2 a
24-A-2	284.3 ± 3.1 c	399.6 ± 1.7 b	111.1 ± 3.0 b	21.4 ± 0.7 c	3.0 ± 0.1 ab	49.7 ± 0.5 b	36.3 ± 1.8 c	684.6 ± 3.7 b	795.0 ± 5.2 b
24-A-3	293.1 ± 3.0 b	377.1 ± 1.9 c	108.5 ± 2.3 b	22.0 ± 0.5 bc	3.2 ± 0.2 ab	50.5 ± 1.3 b	42.8 ± 1.2 b	660.3 ± 3.8 c	778.7 ± 3.7 c
24-B-1	245.1 ± 0.4 f	283.3 ± 5.2 g	93.2 ± 1.3 c	15.9 ± 0.4 e	3.0 ± 0.2 ab	34.5 ± 5.5 d	27.4 ± 0.6 e	540.9 ± 1.8 f	621.6 ± 5.3 f
24-B-2	284.7 ± 0.9 c	338.7 ± 1.6 e	92.8 ± 1.2 c	22.7 ± 1.0 b	3.2 ± 0.6 ab	45.9 ± 0.8 c	30.9 ± 1.4 d	613.6 ± 2.2 d	716.2 ± 1.1 d
24-B-3	275.9 ± 2.8 d	345.7 ± 4.4 d	90.8 ± 1.4 cd	22.4 ± 0.8 bc	2.8 ± 0.1 b	43.7 ± 0.7 c	24.6 ± 0.5 f	618.9 ± 5.5 d	712.5 ± 5.7 d
24-C-1	255.4 ± 3.2 e	310.0 ± 4.3 f	81.5 ± 0.5 d	18.0 ± 0.8 d	2.8 ± 0.0 b	37.0 ± 0.4 d	24.0 ± 0.5 f	565.1 ± 0.5 e	646.9 ± 1.5 e
24-C-2	182.8 ± 0.5 g	259.4 ± 1.1 i	57.7 ± 0.5 f	10.3 ± 0.1 g	1.4 ± 0.0 d	23.5 ± 0.1 f	16.2 ± 0.2 h	448.4 ± 0.7 h	499.9 ± 0.5 h
24-C-3	183.9 ± 3.8 g	269.7 ± 2.4 h	70.1 ± 0.8 e	11.4 ± 0.5f	2.0 ± 0.0 c	27.5 ± 0.8 e	20.5 ± 0.8g	462.4 ± 1.9 g	523.7 ± 1.1 g
2023									
23-A-1	84.8 ± 1.3 c	77.5 ± 0.8 c	30.6 ± 0.3 c	6.5 ± 0.2 c	0.9 ± 0.0 d	15.1 ± 0.3 c	7.9 ± 0.1 d	162.5 ± 0.6 c	192.9 ± 0.8 c
23-B-1	120.0 ± 1.2 b	90.7 ± 2.0 b	38.6 ± 0.8 b	9.2 ± 0.4 b	1.6 ± 0.0 a	20.3 ± 0.3 b	14.5 ± 0.3 a	203.7 ± 0.8 b	249.3 ± 1.0 b
23-B-2	155.2 ± 0.8 a	118.7 ± 0.9 a	58.8 ± 0.8 a	10.5 ± 0.1 a	1.5 ± 0.0 b	27.2 ± 0.6 a	14.2 ± 0.1 b	279.5 ± 1.4 a	332.8 ± 0.6 a
23-C-1	80.9 ± 0.2 d	64.2 ± 0.4 d	30.1 ± 0.2 d	4.2 ± 0.1 d	1.0 ± 0.0 c	11.8 ± 0.1 d	12.0 ± 0.1 c	146.2 ± 0.2 d	175.2 ± 0.4 d
2021									
21-A-1	56.2 ± 0.6 b	42.3 ± 0.5 c	21.1 ± 0.4 b	2.8 ± 0.2 b	0.6 ± 0.0 b	8.1 ± 0.1 b	6.3 ± 0.6 b	101.8 ± 0.9 b	119.6 ± 1.4 b
21-A-2	75.8 ± 0.6 a	60.5 ± 0.9 a	27.9 ± 1.5 a	3.9 ± 0.1 a	1.0 ± 0.0 a	10.7 ± 0.3 a	11.9 ± 0.3 a	136.6 ± 2.4 a	164.2 ± 2.5 a
21-B-2	38.2 ± 0.5 d	53.6 ± 1.4 b	11.1 ± 0.3 c	2.4 ± 0.3 c	0.5 ± 0.0 c	4.4 ± 0.6 c	4.9 ± 0.3 c	90.8 ± 0.2 c	103.0 ± 1.5 c
21-C-1	76.0 ± 0.2 a	59.4 ± 0.7 a	27.5 ± 0.2 a	3.7 ± 0.2 a	1.0 ± 0.0 a	10.0 ± 0.6 a	11.2 ± 0.1 a	137.1 ± 0.0 a	163.0 ± 0.7 a
21-C-2	40.9 ± 0.5 c	36.4 ± 0.4 d	17.7 ± 0.2 b	2.1 ± 0.0 c	0.4 ± 0.0 d	5.2 ± 0.0 c	4.2 ± 0.5 c	83.3 ± 0.6 d	95.1 ± 1.1 d
2019									
19-A-1	41.1 ± 0.4 a	35.1 ± 1.5 a	14.6 ± 0.5 a	2.5 ± 0.1 a	0.6 ± 0.0 a	5.6 ± 0.5 a	7.4 ± 1.0 a	74.7 ± 0.8 a	90.8 ± 2.3 a
19-B-1	37.5 ± 0.4 c	35.1 ± 0.3 a	13.7 ± 0.4 ab	2.2 ± 0.0 b	0.6 ± 0.0 a	5.3 ± 0.2 a	7.5 ± 0.3 a	70.8 ± 0.5 b	86.3 ± 0.5 b
19-B-2	29.4 ± 0.3 d	28.4 ± 0.4 b	12.1 ± 0.1 bc	1.6 ± 0.0 c	0.4 ± 0.0 b	4.2 ± 0.1 b	4.1 ± 0.1 b	59.6 ± 0.6 c	69.9 ± 0.6 c
19-C-1	38.5 ± 0.4 b	34.8 ± 0.8 a	14.0 ± 0.1 ab	2.2 ± 0.0 b	0.6 ± 0.0 a	5.4 ± 0.1 a	7.8 ± 0.1 a	71.3 ± 0.8 b	87.3 ± 0.9 b
19-C-2	26.9 ± 0.2 e	27.0 ± 0.2 c	11.3 ± 0.5 c	1.5 ± 0.1 d	0.3 ± 0.0 c	3.6 ± 0.0 c	4.0 ± 0.1 b	55.7 ± 0.6 d	65.1 ± 0.6 d

Note: Data are presented as ‘mean ± standard deviation’. Different letters within the same column for the same year indicate significant differences (*p* < 0.05).

**Table 2:** The content of nonanthocyanin phenolic compounds in wines.

Sample	Isorham -Netin	Myricetin	Quercetin	Kaemp -Ferol	Syringe -Tin	Flavonols	Catechin	Epicate -Chin	Gallocate -Chin	Epigallo -Catechin	Procya -Nin B1	Procya -Nin B2	Flavan-3-ols
2024													
24-A-1	53.1 ± 1.3 f	69.0 ± 2.0 e	35.4 ± 1.8 c	1.8 ± 1.6 a	42.2 ± 0.5 a	201.5 ± 4.8 d	58.6 ± 0.2 g	32.6 ± 0.3 c	4.9 ± 0.0 bc	2.3 ± 0.0 a	11.9 ± 0.1 c	6.8 ± 0.0 ab	117.1 ± 0.2 b
24-A-2	71.3 ± 0.8 b	75.3 ± 0.7 c	40.7 ± 0.5 b	1.1 ± 1.9 a	41.8 ± 0.3 ab	230.2 ± 3.1 b	41.0 ± 0.7 h	24.2 ± 0.7 d	4.1 ± 0.1 d	1.4 ± 0.0 d	12.9 ± 0.9 ab	6.7 ± 0.5 ab	111.3 ± 16.8 b
24-A-3	80.3 ± 0.1 a	120.5 ± 0.5 a	23.6 ± 1.0 e	1.7 ± 0.0 a	40.9 ± 0.7 bc	267.0 ± 1.4 a	64.1 ± 0.3 b	32.5 ± 0.3 c	3.3 ± 0.0 f	1.6 ± 0.0 c	9.3 ± 0.1 d	3.7 ± 0.0 f	114.6 ± 0.0 b
24-B-1	59.2 ± 0.3 d	99.1 ± 0.3 b	9.4 ± 0.4 f	1.5 ± 0.1 a	41.0 ± 0.8 bc	210.2 ± 1.1 c	72.4 ± 0.6 a	36.4 ± 0.9 a	3.7 ± 0.0 e	1.7 ± 0.0 b	11.0 ± 0.0 e	4.5 ± 0.1 e	129.7 ± 0.5 a
24-B-2	63.5 ± 0.4 c	70.9 ± 0.6 d	33.9 ± 0.5 c	2.3 ± 0.1 a	40.4 ± 0.3 c	211.0 ± 0.7 c	61.1 ± 0.6 de	33.7 ± 0.4 b	4.9 ± 0.1 bc	1.5 ± 0.1 d	13.2 ± 0.2 a	7.0 ± 0.1 a	121.3 ± 1.0 ab
24-B-3	56.5 ± 0.3 e	74.1 ± 0.7 c	42.9 ± 0.8 a	1.6 ± 0.1 a	36.2 ± 0.8 d	211.3 ± 0.7 c	60.2 ± 1.3 ef	31.7 ± 0.7 c	6.3 ± 0.1 a	1.7 ± 0.0 b	12.6 ± 0.0 b	6.5 ± 0.0 bc	119.0 ± 0.7 b
24-C-1	37.2 ± 0.8 g	38.9 ± 0.9 h	22.3 ± 1.0 e	1.0 ± 0.3 a	28.1 ± 0.4 g	127.5 ± 3.2 f	59.3 ± 0.4 fg	31.6 ± 0.5 c	4.9 ± 0.0 c	1.5 ± 0.0 d	12.7 ± 0.1 ab	6.2 ± 0.0 cd	116.1 ± 0.3 b
24-C-2	36.5 ± 0.4 g	40.7 ± 0.5 g	22.8 ± 0.2 e	1.4 ± 0.1 a	29.6 ± 0.2 f	131.0 ± 0.2 f	61.4 ± 0.2 cd	31.8 ± 0.4 c	5.0 ± 0.0 bc	1.4 ± 0.0 e	12.9 ± 0.1 ab	6.3 ± 0.0 cd	118.7 ± 0.6 b
24-C-3	53.3 ± 0.3 f	52.6 ± 0.5 f	28.5 ± 0.4 d	0.8 ± 0.0 a	31.1 ± 0.3 e	166.3 ± 1.0 e	62.2 ± 0.2 c	32.4 ± 0.7 c	5.0 ± 0.0 bc	1.4 ± 0.0 e	11.3 ± 0.0 d	6.1 ± 0.1 d	118.3 ± 0.6 b
2023													
23-A-1	39.5 ± 0.9 a	27.1 ± 0.5 b	17.7 ± 0.1 a	-	7.4 ± 0.1 a	91.7 ± 1.1 a	15.4 ± 0.5 d	10.5 ± 0.1 b	2.9 ± 0.0 c	0.4 ± 0.1 c	3.1 ± 0.0 d	1.1 ± 0.0 c	33.3 ± 0.5 d
23-B-1	24.1 ± 0.7 c	24.0 ± 1.1 c	9.9 ± 0.4 c	-	4.2 ± 0.0 a	62.2 ± 2.1 c	19.3 ± 0.3 c	9.7 ± 0.0 c	3.7 ± 0.1 a	1.2 ± 0.1 a	3.3 ± 0.2 c	1.2 ± 0.1 b	38.4 ± 0.4 c
23-B-2	30.6 ± 0.3 b	30.0 ± 0.4 a	12.2 ± 0.1 b	-	3.9 ± 3.7 a	76.7 ± 4.1 b	22.2 ± 0.5 a	11.6 ± 0.1 a	3.4 ± 0.2 b	1.3 ± 0.1 a	3.8 ± 0.0 b	1.2 ± 0.0 b	43.5 ± 0.7 a
23-C-1	20.2 ± 0.7 d	21.4 ± 0.9 d	4.9 ± 0.3 d	0.1 ± 0.0 a	6.2 ± 0.1 a	52.8 ± 1.3 d	21.4 ± 0.2 b	10.7 ± 0.2 b	2.8 ± 0.0 c	1.1 ± 0.0 b	4.3 ± 0.1 a	1.5 ± 0.0 a	41.7 ± 0.1 b
2021													
21-A-1	35.6 ± 0.5 c	27.4 ± 0.3 b	11.3 ± 0.4 c	-	6.7 ± 0.3 ab	81.0 ± 0.6 d	14.6 ± 0.1 c	8.1 ± 0.1 b	2.4 ± 0.1 bc	0.7 ± 0.1 c	-	2.6 ± 0.0 a	28.5 ± 0.2 c
21-A-2	34.9 ± 0.4 d	30.3 ± 0.4 a	11.6 ± 0.4 c	-	6.0 ± 0.1 b	82.8 ± 0.4 c	13.8 ± 0.1 d	7.7 ± 0.1 d	2.3 ± 0.1 c	0.9 ± 0.0 b	2.1 ± 0.0 d	0.5 ± 0.0 c	27.3 ± 0.1 e
21-B-2	36.8 ± 0.4 b	29.6 ± 0.4 a	11.5 ± 0.4 c	-	7.4 ± 1.0 a	85.3 ± 1.0 b	13.8 ± 0.1 b	7.9 ± 0.1 c	2.4 ± 0.0 bc	0.9 ± 0.0 b	2.3 ± 0.1 c	0.5 ± 0.0 c	27.7 ± 0.2 d
21-C-1	36.2 ± 0.1 b	25.3 ± 0.4 c	14.0 ± 0.6 b	-	6.2 ± 0.2 b	81.7 ± 0.7 cd	16.9 ± 0.1 a	10.4 ± 0.0 a	2.4 ± 0.1 b	1.0 ± 0.1 a	2.8 ± 0.1 b	1.1 ± 0.1 b	34.6 ± 0.1 a
21-C-2	38.1 ± 0.1 a	26.7 ± 0.5 b	15.7 ± 0.7 a	-	6.2 ± 0.1 b	86.7 ± 0.6 a	16.2 ± 0.1 b	10.3 ± 0.1 a	2.9 ± 0.0 a	0.5 ± 0.0 d	3.0 ± 0.0 a	-	32.9 ± 0.2 b
2019													
19-A-1	39.5 ± 0.1 a	30.2 ± 0.3 a	12.1 ± 0.5 b	-	7.3 ± 0.4 a	89.1 ± 1.2 a	14.5 ± 0.1 b	8.4 ± 0.3 c	3.2 ± 0.1 a	0.9 ± 0.0 a	2.5 ± 0.0 b	0.7 ± 0.0 b	30.3 ± 0.2 c
19-B-1	32.6 ± 0.1 e	23.9 ± 0.4 d	13.5 ± 0.6 a	-	6.8 ± 0.1 d	76.8 ± 0.4 d	15.2 ± 0.1 a	10.3 ± 0.1 b	2.9 ± 0.1 bc	1.0 ± 0.1 a	2.2 ± 0.2 c	0.8 ± 0.1 ab	32.4 ± 0.3 b
19-B-2	35.5 ± 0.1 c	25.9 ± 0.3 c	12.4 ± 0.3 b	-	5.1 ± 0.1 b	78.9 ± 0.1 c	14.5 ± 0.0 b	10.5 ± 0.2 b	2.9 ± 0.1 bc	1.0 ± 0.0 a	2.6 ± 0.0 b	0.8 ± 0.2 ab	32.3 ± 0.3 b
19-C-1	33.6 ± 0.1 d	24.2 ± 0.2 d	10.6 ± 0.7 c	-	5.7 ± 0.1 c	74.1 ± 0.7 e	15.3 ± 0.1 a	10.9 ± 0.1 a	3.0 ± 0.1 b	1.0 ± 0.1 a	2.7 ± 0.0 ab	0.9 ± 0.0 a	33.8 ± 0.3 a
19-C-2	36.7 ± 0.0 b	27.2 ± 0.3 b	12.0 ± 0.4 b	-	6.7 ± 0.3 b	82.6 ± 0.6 b	15.3 ± 0.1 a	10.8 ± 0.1 a	2.8 ± 0.0 c	1.0 ± 0.1 a	2.7 ± 0.0 a	0.9 ± 0.1 a	33.6 ± 0.2 a

Note: Data are presented as ‘mean ± standard deviation’. Different letters within the same column for the same year indicate significant differences (*p* < 0.05).

## Data Availability

The original contributions presented in the study are included in the article, further inquiries can be directed to the corresponding author.

## Appendix A

**Table A1:** Basic Physicochemical Parameters of Wine.

Sample	Volatile Acid (g/L)	Total Acid (g/L)	pH	Alcoholic Strength (%Vol)	Leftovers (g/L)
2024					
24-A-1	0.43 ± 0.09 d	5.79 ± 0.3 b	3.79 ± 0.03 b	13.68 ± 0.18 b	4.80 ± 0.13 cd
24-A-2	0.59 ± 0.07 bc	6.11 ± 0.09 a	3.79 ± 0.04 b	13.65 ± 0.13 bc	4.97 ± 0.24 bc
24-A-3	0.70 ± 0.06 ab	6.15 ± 0.11 a	3.73 ± 0.02 c	12.91 ± 0.11 f	5.38 ± 0.15 a
24-B-1	0.65 ± 0.05 ab	5.48 ± 0.26 c	3.82 ± 0.02 ab	15.21 ± 0.11 a	5.15 ± 0.07 ab
24-B-2	0.73 ± 0.10 a	5.45 ± 0.20 c	3.81 ± 0.05 b	15.13 ± 0.17 a	4.49 ± 0.14 e
24-B-3	0.69 ± 0.11 ab	6.26 ± 0.09 a	3.80 ± 0.02 b	13.62 ± 0.16 bc	5.17 ± 0.14 ab
24-C-1	0.65 ± 0.01 abc	6.34 ± 0.13 a	3.79 ± 0.01 b	13.12 ± 0.19 de	5.28 ± 0.03 a
24-C-2	0.63 ± 0.03 abc	6.18 ± 0.16 a	3.87 ± 0.05 a	13.02 ± 0.22 de	5.14 ± 0.09 ab
24-C-3	0.52 ± 0.05 cd	6.30 ± 0.08 a	3.80 ± 0.02 b	13.33 ± 0.31 cd	4.61 ± 0.19 de
2023					
23-A-1	0.64 ± 0.03 a	5.50 ± 0.18 a	3.70 ± 0.09 a	14.13 ± 0.12 a	5.25 ± 0.06 b
23-B-1	0.54 ± 0.01 b	5.44 ± 0.10 a	3.87 ± 0.05 b	11.49 ± 0.14 b	5.68 ± 0.14 a
23-B-2	0.46 ± 0.03 c	5.49 ± 0.24 a	3.96 ± 0.04 a	12.54 ± 0.06 c	3.58 ± 0.09 c
23-C-1	0.64 ± 0.02 a	5.43 ± 0.36 a	3.71 ± 0.04 b	12.71 ± 0.21 b	5.4 ± 0.10 b
2021					
21-A-1	0.56 ± 0.09 b	5.57 ± 0.22 b	3.64 ± 0.06 a	14.08 ± 0.18 b	5.15 ± 0.18 c
21-A-2	0.77 ± 0.06 a	5.95 ± 0.18 a	3.67 ± 0.04 a	14.53 ± 0.23 a	5.90 ± 0.24 a
21-B-2	0.65 ± 0.11 ab	5.50 ± 0.31 b	3.66 ± 0.04 a	13.96 ± 0.16 b	5.36 ± 0.14 bc
21-C-1	0.61 ± 0.05 b	5.44 ± 0.05 b	3.73 ± 0.06 a	12.69 ± 0.19 c	5.52 ± 0.09 b
21-C-2	0.53 ± 0.08 b	5.39 ± 0.15 b	3.70 ± 0.04 a	12.57 ± 0.17 c	5.56 ± 0.19 b
2019					
19-A-1	0.54 ± 0.07 bc	5.18 ± 0.08 a	3.75 ± 0.03 a	14.23 ± 0.13 a	5.31 ± 0.27 a
19-B-1	0.45 ± 0.05 c	5.39 ± 0.16 a	3.59 ± 0.07 b	12.94 ± 0.14 b	4.00 ± 0.19 b
19-B-2	0.63 ± 0.04 ab	5.27 ± 0.07 a	3.79 ± 0.01 a	12.72 ± 0.32 bc	4.33 ± 0.22 b
19-C-1	0.46 ± 0.02 c	5.35 ± 0.19 a	3.75 ± 0.05 a	12.36 ± 0.16 d	4.06 ± 0.26 b
19-C-2	0.65 ± 0.06 a	5.33 ± 0.12 a	3.71 ± 0.07 a	12.50 ± 0.09 cd	4.38 ± 0.17 b

Note: Data are presented as ‘mean ± standard deviation’. Different letters within the same column for the same year indicate significant differences (*p* < 0.05).
